# CETA and pharmaceuticals: impact of the trade agreement between Europe and Canada on the costs of prescription drugs

**DOI:** 10.1186/1744-8603-10-30

**Published:** 2014-05-06

**Authors:** Joel Lexchin, Marc-André Gagnon

**Affiliations:** 1 School of Health Policy and Management, York University Ontario, Toronto, Ontario M3J 1P3, Canada; 2 University Health Network, Toronto, Canada; 3 Department of Family and Community Medicine, University of Toronto, Toronto, Ontario, Canada; 4 School of Public Policy and Administration, Carleton University, Ottawa, Ontario, Canada

**Keywords:** Brand-name drugs, Canada, CETA, Data protection, European Union, Intellectual Property rights, Patent linkage, Patent term restoration

## Abstract

On a per capita basis, Canadian drug costs are already the second highest in the world after the United States and are among the fastest rising in the Organization for Economic Co-Operation and Development. The Comprehensive Economic and Trade Agreement (CETA) between the European Union (EU) and Canada will further exacerbate the rise in costs by:

• Committing Canada to creating a new system of patent term restoration thereby delaying entry of generic medicines by up to two years;

• Locking in Canada’s current term of data protection, and creating barriers for future governments wanting to reverse it;

• Implementing a new right of appeal under the patent linkage system that will create further delays for the entry of generics.

CETA will only affect intellectual property rights in Canada—not the EU. This analysis estimates that CETA’s provisions will increase Canadian drug costs by between 6.2% and 12.9% starting in 2023. The Canadian government committed to compensating provinces for the rise in costs for their public drug plans. Importantly, this means that people paying out-of-pocket for their drugs or receiving them through private insurance, will be charged twice: once through higher drug costs and once more through their federal taxes.

As drug costs continue to grow, there are limited options available for provincial/territorial governments: restrict the choice of medicines in public drug plans; transfer costs to patients who typically are either elderly or sick; or take money from other places in the health system, and threaten the viability of Canada’s single payer system. CETA will therefore negatively impact the ability of Canada to offer quality health care.

## Background

Negotiations for the Comprehensive Economic and Trade Agreement (CETA) between Canada and the European Union were launched in May 2009. In October 2013, the negotiating parties announced they had reached an agreement in principle over CETA and that its implementation should begin in 2015. One of the most controversial issues about CETA is its proposal to extend intellectual property protection for patented drugs in Canada, which could significantly increase drug costs for Canadians. We recognize that trade deals are complex and involve trade-offs for other benefits that countries hope to obtain. In this article we are not attempting to weigh the overall benefits and costs to Canada of CETA, rather we are just focusing on the impact that CETA will have on expenditures for patented prescription drugs.

At over $700 per person per year (US$ purchasing power parity), Canada spends more per capita on pharmaceuticals than any other country in the world except the United States (US) [1]. Similarly when measured against comptor countries in the Organization for Economic Cooperation and Development (OECD), Canada’s growth in drug spending per capita (in real terms) between 2000 to 2009 was 4.3 percent per year compared to the OECD average of 3.5 percent. Although this rate fell to -0.3 percent per year from 2009–2011, the OECD average fell to -0.9 percent [1]. Canada represented 2.6% of the global market sales in prescription drugs in 2011, while the United Kingdom, with a population almost twice as large, made up only 2.5% of the global market [2]. In 2012 Canada was spending almost as much on prescription drugs at $27.73 billion as it was on physicians at $29.96 billion [3].

Compared to other health expenditures, from 1985 to 2006, Canadian drug spending consistently grew at a faster rate than overall health spending [4]. There were a number of cost drivers, including population growth and aging, general inflation, price effects (the cost of purchasing an individual drug), volume effects (number and size of prescriptions) and mix effects (changes in the drugs selected to treat a particular condition). Although population growth and aging are often cited as major reasons for spending increases, in fact the second largest contributor, after volume effects, was mix effects, i.e., substituting newer, more expensive drugs for older, less expensive ones [5]. While using more expensive drugs is justified when they are therapeutically superior, overall fewer than 1 in 10 new drugs offer any significant therapeutic advantages [6].

Since 2007, the growth in drug spending has slowed and in 2011 and 2012 was 3.8% and 3.2%, respectively [7]. The trend to slower growth is arguably due to a combination of two factors: the expiration of patents on blockbuster drugs (also known as the patent cliff) alongside the subsequent entry of lower priced generics, and the move in a number of Canadian provinces to lower generic prices [7]. The impact of such provincial policy changes is seen through Ontario’s expenditure on atorvastatin (Lipitor) – a drug used to treat high cholesterol. In 2009–10, prior to patent expiration, this medication cost Ontario $316 million [8]. Once Lipitor’s patent expired and generics were available, the cost for atorvastatin dropped in 2010–11 to $133 million [9], thereby saving Ontario $183 million. These savings will increase as provinces aggressively lower the price that they pay for generics as Ontario, British Columbia and Alberta, among other provinces, have done within the past few years [10].

This paper discusses the three main provisions in CETA that relate to patented drugs and explains how they will lead to increased drugs costs.

## Discussion

### Impact of CETA’s intellectual property rights provisions

By understanding Canada’s current pharmaceutical regime, we can better explore the potential impact that will be felt through CETA—specifically its intellectual property rights provisions (IPRs). There are three provisions affecting IPRs in CETA that pose a serious threat to the anticipated savings from generic drugs like those seen in Ontario: patent term restoration, a consolidation of data protection, and a right of appeal under the Notice of Compliance (NOC) regulations. The following section explores the rationale behind each of these provisions and explains how their implementation will negatively impact Canada’s capacity to control drug costs.

#### *Patent term restoration*

Under the terms of the World Trade Organization’s 1994 Agreement on Trade Related Aspects of Intellectual Property Rights (TRIPS), patents on pharmaceuticals – and all other goods – run for 20 years from the time that the patent application is filed. CETA will now allow for what is called a “*sui generis* protection” (also called “patent term restoration”) that can provide up to two additional years of patent protection. The aim of patent term restoration is to compensate companies for the time lost between when the patent application is filed and when the drug is eventually approved. The period of the patent term restoration will be calculated by taking the time between when the patent was applied for and when the product was marketed and subtracting 5 years. As long as the result is 2 years or less, that additional time will be added to the length of the patent. It seems that this additional time will be available even if the company/patentee is responsible for any delays in the approval process [11]. According to Rx & D, the association representing the Canadian brand-name drug companies, “Canada remains the only developed nation that provides no form of compensation to innovative pharmaceutical companies for regulatory approval delays” [12].

The rationale in support of patent term-restoration is that without such a change, Canada has an incentive to slow down the approval process. A comptive analysis by a multinational law firm regularly consulting for the brand-name pharmaceutical industry, justifies patent term restoration by claiming that Canadian drug approval times are 152 days slower than those in the EU (433 versus 281 days). The report claims that slower drug approval means that drugs launched in Canada may have far less time remaining from the 20-year patent term compared to drugs launched in the EU [13]. The Canadian Generic Pharmaceutical Association commissioned a response to this comptive analysis. In the response, Hollis and Grootendorst find the data used in the comptive analysis problematic in two key elements. “First, while assessment averages 281 days in Europe, the EMA [European Medicines Agency] report clearly states that *approval takes an additional 79 days* beyond the assessment period.**..** Compounding this error, almost half of the EMA approvals were for ‘generic or hybrid medicines and informed consent applications’ which are obviously very different in nature from the New Drug Submissions in the Canadian data” [14]. In addition, the longer approval time in Canada is the result of four specific drugs where the initial regulatory submission from the company was deemed deficient or non-compliant with Health Canada’s filing requirements and more information was requested from the manufacturer. There was a prolonged delay before drug companies finally submitted the required information, thereby artificially inflating the average difference between Canada and the EU. When these four drugs were excluded from the calculations, Canadian approval times for the remaining drugs are on average 67 days less than in Europe [14].

Finally, there is an additional serious error in the Rx & D sponsored analysis. The report only compares approval times for 22 drugs. When using a larger sample of drugs approved by either the EMA or Health Canada between 2001 and 2010, the median approval time in Europe was 366 days (interquartile range, 310 to 447) and 393 days (interquartile range, 310 to 603) at Health Canada, for a difference of 27 days instead of 152. Finally, when considering drugs approved by both the European Medicines Agency and Health Canada, that difference drops to just 10 days [15].

#### *Data protection*

In addition to patent term restoration, another provision of CETA that will put pressure on drug costs pertains to the data protection provisions of CETA. The “data” in this term refers to the safety and efficacy information that brand-name companies generate through the clinical trials they conduct in order to get drugs approved. Typically generic companies rely on this data when they submit applications to get products approved. Both the North American Free Trade Agreement (NAFTA) and the TRIPS agreement specify that data should be protected for five years although even that five-year period is subject to interpretation. Article 39.3 in TRIPS only requires countries to protect against “unfair commercial use” of marketing approval data but gives countries considerable discretion to define “unfair” in the context of their own national laws and culture. “Countries can meet their obligations to protect against “unfair commercial use” under Article 39.3 by barring “dishonest” uses of test data. Countries are not obligated under Article 39.3 to confer exclusive rights on the originator of marketing approval data [16].

In 2006, Canada extended data protection to eight years of market exclusivity with an additional six months if companies have studied the drug in a pediatric population. Generic companies are not allowed to make use of the brand-name companies’ data in their applications for a minimum of six years [17]. Although CETA will not extend data protection, Canada has “agreed to lock in the current Canadian practice of providing eight years of market exclusivity” [18], making it virtually impossible for any future government to shorten the period.

Moreover, up until CETA, data protection was only granted to new chemical entities, i.e., drugs that have never been sold in any form in Canada. During the negotiations, the EU was demanding that improved data protection be granted for any pharmaceutical product rather than just new chemical entities. Limited information about the contents of CETA makes it unclear if the range of products available for eight years of data protection will be expanded to include products representing minor changes to an existing drug [11]. If the EU’s demand has been accepted, the net effect would be to effectively offer financial incentives for companies to engage in minor molecular manipulation and produce drugs that offer no new therapeutic advances.

#### *Right of appeal*

The final CETA provision regarding IPR that will put pressure on drug costs by delaying the appearance of generics, is the right of appeal. A Notice of Compliance (NOC) is the term Health Canada uses when it certifies that a drug manufacturer has met Health Canada's regulatory requirements for the safety, efficacy and quality of a product. In 1993, the federal government introduced the NOC linkage regulations as part of the legislation that abolished compulsory licensing to import generic drugs into Canada. Under the linkage regulations Health Canada is prevented from issuing an authorization for market entry for a generic until the generic company can show that all of the relevant patents on the brand name product have expired. As a result, when the generic company submits its application to get a product approved it also sends a Notice of Allegation (NOA) to the patent holder claiming that no patents are being infringed. The patent holder then has 45 days in which to initiate an application in the Federal Court of Canada seeking an order to prohibit Health Canada from issuing a NOC to the generic manufacturer for a period of up to 24 (originally 30) months. At that point, the matter usually proceeds to a court hearing. The stay expires either at the end of the 24 months, when the disputed patent expires or when the court case is decided, whichever comes first [19].

The argument put forward by the brand-name industry has been that if the generic company wins the court case and is allowed to market its product, then once a NOC has been issued any appeal filed by the patentee becomes moot [20]. The patentee is thus left with no alternative but to start another proceeding (an action for patent infringement) once the generic has entered the market. CETA will now allow brand-name companies the right to appeal decisions made under the NOC linkage regulations. However, the generic companies have received written assurances from the Government of Canada that its implementation of the “Right of Appeal” treaty commitment will also address excessive and duplicative litigation by ending the practice of dual litigation. Dual litigation means that even if brand-name companies lose under the NOC linkage regulations, they can launch a septe case under Canada’s general patent law. It is this ability to launch a second court case that the federal government has pledged to end. Although this will work to the advantage of the generic companies there will still potentially be an additional delay to the marketing of generic drugs.

Since the EU does not use patent linkage and CETA does not require it to do so, this Right of Appeal provision only applies to Canada. The EU currently allows interlocutory injunctions that prevent a generic from launching until the litigation is complete or the parties have settled. The ease of obtaining such injunctions varies according to each national jurisdiction and it has been argued that these injunctions are the equivalent of Canada’s NOC regulations [21]. Patent linkage systems, however, automatically deliver the equivalent of an injunction without prior analysis of evidence that a patent is being infringed [22]. In fact, the European Commission prohibits EU member countries from introducing patent linkage provisions because they delay the entry of generics. Italy was reprimanded in 2012 for trying to introduce such a system and was asked to eliminate it [23]. It is thus ironic that under CETA, rather than Canada eliminating its patent linkage system, it will be forced to strengthen it by providing a right of appeal that will create further delays for the entry of generics. In practice, this means that under CETA there could be a further delay of 6–18 months before generics appear, as the appeal makes its way through the court system [11].

### Financial implications of CETA

Although it is impossible to be sure what the final financial implications of CETA will be once its IPR provisions fully come into effect, Grootendorst and Hollis used the sample of the 15 drugs for which a generic appeared on the Canadian market in 2010 to provide an estimate of what would have been the consequences if all of CETA’s provisions were fully implemented in 2010. The analysis of the impact of the different provisions was performed by Gilbert’s LLP, a law firm specializing in patent litigation. The result of the analysis, undertaken before the CETA negotiations were completed, was that CETA would delay the entry of generics by 3.46 years on average and that the annual loss for every additional year of entry delay was $811 million, leading to an additional cost of $2.8 billion per year [11].

Internal documents from the federal government also estimated that the additional costs for patented drugs could be up to $2 billion [24], but the methodology used to arrive at this estimate is not known and Trade Minister Ed Fast has refused to release these government documents [25].

The model used by Hollis and Grootendorst included delays due to the right of appeal under NOC regulations, extension of data exclusivity, and implementation of a patent term restoration of a maximum of five years (plus an additional six months when pediatric trials were conducted). We have revised their calculations to adjust them for the actual clauses found in CETA, i.e., right of appeal under NOC regulations and patent term restoration of a maximum two years. If we use the same sample of 15 drugs, and if we assume that data exclusivity is only extended to innovative drugs, we observe that if CETA was fully implemented in 2010 (the reference year for the Hollis and Grootendorst analysis), it would have increased the average market exclusivity for patented drugs by 358.4 days, or 0.98 years, which would bring an additional yearly cost of $795 million, or 6.2% of the total annual cost of patented drugs, which was $12.8 billion for that year [2]. If CETA extends data exclusivity to non-innovative drugs, the average delay would increase by 741 days, or 2.03 years, which represents an additional yearly cost of $1,645 million, or 12.9% of total costs of patented drugs. [For the details of the calculations, see Additional file 1: Table S1 and S2] While we believe that our update of the calculations done by Grootendorst and Hollis represents a reasonable estimate of the effects of the CETA provisions on Canadian drug expenditures we acknowledge that their figures, based on the analysis performed by the law firm Gilbert’s LLP, have not been verified by any individuals independent of both the generic and brand-name industries.

The additional costs cited above assume that CETA’s provisions are applied to drugs currently on the market. As such they are only approximations of what the eventual costs will be since patent term restoration will only apply to drugs approved after CETA is ratified. Generic equivalents for these drugs will only start to appear around 2023 [26] and the actual additional costs will depend on how many drugs receive patent term restoration and what their sales are. In addition, overall increases in provincial costs could be mitigated if the provincial plans impose price restrictions on patented medications, or implement stricter conditions for listing these products on their formularies. However, even if these measures do occur they will not impact on the costs for people with private insurance or who pay out of pocket.

The federal government has announced that it will compensate provinces for the rise in drug costs for their public drug plans [27]. If this proves to be the case, then instead of Canadian taxpayers paying the additional costs for prescription drugs at the provincial level they will simply pay at the federal level. Importantly, people paying out of pocket for their drugs, or through private insurance, will not benefit from this compensation. Estimates are that 13% of the Canadian population is either uninsured or underinsured for prescription drug costs [28] and that cost-related nonadherence is 35% among people with low income and no insurance [29]. People with no drug coverage and paying out of pocket are usually people with minimum wage jobs [28] and are often the least able to absorb increases in prices. No compensation will be given for either co-payments or deductibles paid out-of-pocket by insured patients covered by a public drug plan. Therefore, whatever compensatory measures the federal government is committing to, in order to help provinces offset the predicted cost increases, will not help those who will be the most impacted by these increases.

## Conclusion

If drug costs continue to grow, there are limited options for cost containment and most of them would involve greater hardship for people who need the medications: restrict the choice of medicines that the provinces offer to their citizens; place more of the burden of costs on individuals, typically the elderly and the sick; or take money out of other places in the health system, thereby threatening the viability of Canada’s single payer system. Canadians should not have to accept any of these choices. Instead, if the growth in costs is inevitable then we could consider raising taxes. While we see that as a viable option it is highly unlikely to be taken up by provincial and federal governments. Finally, measures could be taken to ensure that costs are controlled. One of those measures is rejecting the parts of CETA that will significantly impact the ability of Canadians to afford quality health care.

## Competing interests

In 2007 Joel Lexchin was a consultant to a law firm acting for Apotex Inc. In 2008 he was an expert witness for the Canadian federal government in its defence against a lawsuit challenging the ban on direct-to-consumer advertising. In 2010 he was an expert witness for a law firm representing the family of a plaintiff who allegedly died from an adverse reaction from a product made by Allergan. He is currently on the Management Board of Healthy Skepticism Inc. and is the Chair of the Health Action International – Europe Association Board.

Marc-André Gagnon reports no competing interests.

## Authors’ contribution

JL and MAG jointly conceived of the idea for this paper, gathered and analyzed the data and wrote and revised the paper. Both authors read and approved the final manuscript.

## Supplementary Material

Additional file 1: Table S1 Potential delays, in days, for generic entry in Canada if all Europeans demands for inclusion in CETA were met. **Table S2.** Potential delays, in days, for generic entry in Canada based on announced CETA provisions. Click here for file

## References

[B1] OECD Health at a Glance 2013 OECD Indicators 2013

[B2] Patented Medicine Prices Review Board Annual report 2012 2013 Ottawa: PMPRB

[B3] Canadian Institute for Health Information National health expenditure trends, 1975 to 2012 2013 Ottawa: CIHI

[B4] Canadian Institute for Health Information Drug expenditure in Canada, 1985 to 2011 2012 Ottawa: CIHI

[B5] Canadian Institute for Health Information Drivers of prescription drug spending in Canada 2012 Ottawa: CIHI

[B6] Prescrire Editorial Staff New drugs and indications in 2011: France is better focused on patients’ interests after the Mediator scandal, but stagnation elsewhere Prescrire Int 2012 21 106 110 22515141

[B7] Canadian Institute for Health Information Drug expenditure in Canada, 1985 to 2012 2013 Ottawa: CIHI

[B8] 2009/10 report card for the Ontario Drug Benefit Program Ontario Ministry of Health and Long-Term Care http://www.health.gov.on.ca/en/public/programs/drugs/publications/opdp/docs/odb_report_09.pdf

[B9] 2010/11 report card for the Ontario Drug Benefit Program Ontario Ministry of Health and Long-Term Care http://www.health.gov.on.ca/en/public/programs/drugs/publications/opdp/docs/odb_report_10.pdf

[B10] Canadian Life Health Insurance Association Inc CLHIA report on prescription drug policy: ensuring the accessibility, affordability and sustainability of prescription drugs in Canada 2013 Toronto: CLHIA

[B11] Grootendorst P Hollis A The Canada-European Union Comprehensive Economic & Trade Agreement: an economic impact assessment of proposed pharmaceutical intellectual property provisions J Generic Med 2011 8 81 103 10.1177/1741134311408275

[B12] Canada’s Research-Based Pharmaceutical Companies Reality check: analysis of the CGPA’s economic assessment of proposed pharmaceutical IP provisions 2011 Ottawa: Rx & D

[B13] Kierans P Wall K Daley J CETA trade negotiations 2011 - drug market exclusivity in the EU and Canada 2011 Toronto: Norton Rose

[B14] Hollis A Grootendorst P Drug market exclusivity in the EU and Canada: problems with Norton Rose’s comptive analysis 2012 Toronto

[B15] Downing N Aminawung J Shah N Braunstein J Krumholz H Ross J Regulatory review of novel therapeutics - comparison of three regulatory agencies N Engl J Med 2012 366 2284 2293 10.1056/NEJMsa1200223 22591257 PMC3504361

[B16] Correa C Protection of data submitted for the registration of pharmaceuticals: implementing the standards of the TRIPS agreement 2002 Geneva: The South Centre 15709300

[B17] Government of Canada Regulations amending the Food and Drug Regulations (Data Protection) Can Gazette Part II 2006 140 1493 1502

[B18] Government of Canada Technical summary of final negotiated outcomes: Canada-European Union comprehensive economic and trade deal 2013 Government of Canada

[B19] Faunce T Lexchin J Linkage pharmaceutical evergreening in Canada and Australia Aust N Z Health Policy 2007 4 8 10.1186/1743-8462-4-8 PMC1894804 17543113

[B20] Pharmaceutical Research and Manufacturers of America Special 301 submission 2013 2013 Washington D.C: PhRMA

[B21] Bartucci S Dawson L Pills patents & profits III 2013 Ottawa: MacDonald-Laurier Institute

[B22] Bouchard R Patently innovative: how pharmaceutical firms use emerging patent law to extend monopolies on blockbuster drugs 2011 Cambridge: Biohealthcare Publishing Ltd

[B23] Pharmaceuticals: Commission calls on Italy to comply with EU rules on marketing authorisation of generic drugs 2012 Brussels: European Commission http://europa.eu/rapid/press-release_IP-12-48_en.htm?locale=en

[B24] Canadian Press Canada-EU drug patent demand in trade talks costs almost $2B 2012 CBC News http://www.cbc.ca/news/politics/story/2012/10/15/pol-cp-eu-trade-deal-pharma-costs.html

[B25] Canadian Press Ottawa urged to release papers on drug costs after CETA 2013 CBC News http://www.cbc.ca/news/business/ottawa-urged-to-release-papers-on-drug-costs-after-ceta-1.2418453

[B26] Canada’s Research-Based Pharmaceutical Companies CETA IP changes - impact timeline 2012 Ottawa: Rx & D

[B27] Canada yields on some EU drug IPR demands, but fends off others 2013 Inside US Trade

[B28] Applied Management in association with Fraser Group Tristat Resources Canadians’ access to insurance for prescription medicines: volume 2: the uninsured and the under-insured 2000 Ottawa

[B29] Law M Cheng L Dhalla I Heard D Morgan S The effect of cost on adherence to prescription medications in Canada CMAJ 2012 184 297 302 10.1503/cmaj.111270 22249979 PMC3281154

