# Supplementary material for: CETA and pharmaceuticals: impact of the trade agreement between Europe and Canada on the costs of prescription drugs
Source: Global Health. 2014 May 6;10:30. doi: 10.1186/1744-8603-10-30 (PMC4108121; doi:10.1186/1744-8603-10-30)
Supplement: Additional file 1: Table S1 — Potential delays, in days, for generic entry in Canada if all Europeans demands for inclusion in CETA were met. Table S2. Potential delays, in days, for generic entry in Canada based on announced CETA provisions. [file 1744-8603-10-30-S1.docx]

**Appendix: Details of calculations of potential delays in generic entry as a result of CETA**

**Table S1: Potential delays, in days, for generic entry in Canada if all Europeans demands for inclusion in CETA were met**

| Drug | Data exclusivity increased from 8 to 10 years, including for non-innovative drugs | Right of appeal | Patent term restoration for 5 years | Paediatric exclusivity of 6 months | Total Hollis/Grootendorst |
| --- | --- | --- | --- | --- | --- |
| Actonel | 730 | 0 | 2007 | 184 | 2191 |
| Actonel + Calcium | 2120 | 0 | 1825 | 0 | 2120 |
| Altace HCT | 2353 | 0 | 0 | 0 | 2353 |
| Avandamet | 785 | 0 | 0 | 0 | 785 |
| Cesamet | 0 | 0 | 0 | 0 | 0 |
| Cosopt | 0 | 0 | 0 | 0 | 0 |
| Femara | 0 | 0 | 1846 | 0 | 1846 |
| Gluconorm | 0 | 354 | 0 | 0 | 354 |
| Lipitor | 0 | 0 | 642 | 182 | 824 |
| Primaxin | 1906 | 0 | 0 | 0 | 1906 |
| Proscar | 0 | 0 | 1017 | 0 | 1017 |
| Reminyl ER | 1457 | 365 | 0 | 0 | 1822 |
| Revatio | 2179 | 365 | 0 | 0 | 2179 |
| Strattera | 1560 | 0 | 0 | 0 | 1560 |
| Viramune | 0 | 0 | 0 | 0 | 0 |
| AVERAGE |  |  |  |  | 1264 |

Details of the calculations according to the Hollis and Grootendorst study (2011) based on drugs for which a generic entered the market in 2010. Calculations made by Gilbert’s LLP under the assumption of a patent term restoration of up to five years, additional data exclusivity of two years applied to non-innovative drugs, additional exclusivity of 6 months for paediatric trials and implementation of a right of appeal to NOC regulations.

**Table S2: Potential delays, in days, for generic entry in Canada based on announced CETA provisions**

| Drug | Right of appeal | Patent term restoration for 2 years | No extension of data exclusivity to non-innovative drugs | Data exclusivity including for non-innovative drugs | Extension of data exclusivity for non-innovative drugs |
| --- | --- | --- | --- | --- | --- |
| Actonel | 0 | 730 | 730 | 0 | 730 |
| Actonel + Calcium | 0 | 730 | 730 | 1390 | 1390 |
| Altace HCT | 0 | 0 | 0 | 1623 | 1623 |
| Avandamet | 0 | 730 | 730 | 55 | 730 |
| Cesamet | 0 | 0 | 0 | 0 | 0 |
| Cosopt | 0 | 0 | 0 | 0 | 0 |
| Femara | 0 | 730 | 730 | 0 | 730 |
| Gluconorm | 354 | 0 | 354 | 0 | 354 |
| Lipitor | 0 | 642 | 642 | 0 | 642 |
| Primaxin | 0 | 0 | 0 | 1176 | 1176 |
| Proscar | 0 | 730 | 730 | 0 | 1095 |
| Reminyl ER | 365 | 0 | 365 | 0 | 365 |
| Revatio | 365 | 0 | 365 | 1449 | 1449 |
| Strattera | 0 | 0 | 0 | 830 | 830 |
| Viramune | 0 | 0 | 0 | 0 | 0 |
| AVERAGE |  |  | 359 |  | 741 |

Details of the revised calculations according to the Hollis and Grootendorst study (2011) based on drugs for which a generic entered the market in 2010. Calculations made by Gilbert’s LLP under the assumption of a patent term restoration of up to two years

and implementation of a right of appeal to NOC regulations; with or without data exclusivity extended to non-innovative drugs.
